# Investigation of *SLA4A3* as a candidate gene for human retinal disease

**DOI:** 10.1186/s12952-016-0054-z

**Published:** 2016-05-23

**Authors:** Louise M. Downs, Andrew R. Webster, Anthony T. Moore, Michel Michaelides, Robin R. Ali, Alison J. Hardcastle, Cathryn S. Mellersh

**Affiliations:** Kennel Club Genetics Centre, Animal Health Trust, Newmarket, UK; UCL Institute of Ophthalmology, London, UK; Moorfields Eye Hospital, City Road, London, UK; Present Address: Department of Clinical Studies, School of Veterinary Medicine, University of Pennsylvania, Philadelphia, USA

**Keywords:** SLC4A3, Retinitis pigmentosa, Retinal degeneration

## Abstract

*SLC4A3* has been shown to cause retinal degeneration in a genetically engineered knockout mouse, and in a naturally occurring form of canine progressive retinal atrophy considered to be the equivalent of retinitis pigmentosa in humans (RP). This study was undertaken to investigate if *SLC4A3* coding variants were implicated in human retinal degeneration. *SLC4A3* exons were amplified and sequenced in 200 patients with autosomal recessive retinal degeneration who had no known molecular diagnosis for their condition, which included 197 unrelated individuals with suspected RP and three individuals with other forms of retinal disease. Three rare variants were identified that were predicted to be potentially pathogenic, however each variant was heterozygous in a single patient and therefore not considered disease-causing in isolation. Of these three variants, SNP-3 was the rarest, with an allele frequency of 7.06x10^−5^ (>46,000 exomes from the ExAC database). In conclusion, no compound heterozygous or homozygous potentially pathogenic variants were identified that would account for recessive RP or retinal degeneration in this cohort, however the possibility remains that the rare variants identified could be acting with as yet undiscovered mutations in introns or regulatory regions. *SLC4A3* remains an excellent candidate gene for human retinal degeneration, and with the advent of whole exome and whole genome sequencing of cohorts of molecularly unsolved patients with syndromic and non-syndromic forms of retinal degeneration, *SLC4A3* may yet be implicated in human disease.

**Electronic supplementary material:**

The online version of this article (doi:10.1186/s12952-016-0054-z) contains supplementary material, which is available to authorized users.

## Introduction

*SLC4A3* (solute carrier family 4, member 3; OMIM 106195), encodes the anion exchanger 3 (AE3) protein, which mediates Cl^−^/HCO_3_^−^ exchange across cellular membranes [1]. It is expressed in various tissues including the Müller and horizontal cells of the retina [2], and has been implicated in retinal disease in animals. A knockout mouse model for *Slc4a3* identified *SLC4A3* as a candidate gene for human vitreoretinal degenerations based on their findings of blindness and retinal degeneration in knockout mice [3]. *Slc4a3*^−/−^ mice at four months of age had no gross retinal abnormalities; However, ERG analysis revealed an inner retina defect from birth (reduced b-wave and flicker amplitudes), leading to phototransduction failure at four months (reduced a-wave amplitude). At 4–6 months the number of apoptotic nuclei observed by TUNEL labelling increased. By eight months, pathological signs of photoreceptor degeneration were observed including dense astrocytic processes wrapped around inner retina vessels (a feature analogous to vascular sheathing seen in humans), small diameter major blood vessels, disorganised astrocytic processes at the optic nerve head and rod bipolar cell dendrites aberrantly sprouted into the outer nuclear layer [3].

In addition, we have previously shown that a mutation in *SLC4A3* is associated with a form of naturally occurring autosomal recessive (AR) Progressive Retinal Atrophy (PRA) in the Golden Retriever dog breed, known as GR_PRA1 [4]. We predicted that the homozygous frame-shifting mutation we identified (c.2601_2602insC, predicted to cause a premature stop codon in exon 18, p.E868RfsX104), would result in the loss of a large section of the transmembrane domain and entire C-terminal cytoplasmic domain, including a number of putative carbonic anhydrase binding sites [4]. GR_PRA1 is phenotypically consistent with PRA in other breeds of dog, which is characterized by nyctalopia (night blindness), tapetal hyperreflectivity, retinal vascular attenuation, pigmentary changes and atrophy of the optic nerve head [5].

PRA is widely considered to be the veterinary equivalent of Retinitis Pigmentosa (RP) in humans. RP is the name given to a group of inherited retinal degenerations which affects 1 in 3500 to 4500 people [6]. Photoreceptor cells predominantly affected are the rods and therefore clinical symptoms typically include nyctalopia and loss of peripheral vision. With disease progression, the cones also degenerate resulting in central vision loss and eventually possibly complete blindness [7]. While this heterogeneous group of diseases is highly variable with regard to age of onset, retinal appearance, progression and visual outcome, there are hallmark characteristics secondary to photoreceptor degeneration. These include “bone spicules” caused by pigment granule migration from the retinal pigment epithelium and retinal arteriole and vein attenuation [7]. Inheritance may be AR, autosomal dominant, X-linked (XL) or digenic. With 34 genes implicated to date, AR is the most prevalent mode of inheritance [8]. While PRA is considered the equivalent of RP, due to the limited clinical characterisation of retinal degenerations in dogs, similar retinal diseases can be misdiagnosed as PRA. This suggests that a disease diagnosed as PRA may, in some cases, represent a form of retinal degeneration that is the equivalent of other forms of human retinal dystrophy with similar ophthalmic phenotypes e.g. Leber congenital amaurosis (LCA) or cone-rod dystrophy.

At least 17 naturally occurring dog models with retinal degeneration have been described with equivalent human disease (reviewed in [9]). These are valuable, not only for understanding disease pathology better, but also for developing treatments. For example, mutations in *RPE65* have been implicated in AR retinal degeneration (LCA) in dogs [10, 11] and humans [12, 13], and gene therapy clinical trials have yielded promising results in both species [14–20]. Similarly, mutations in *RPGR* cause XLPRA [21–23] and are responsible for more than 70 % of XLRP cases [24–26]. Gene therapy strategies are underway with promising results in the dog model [27]. Importantly, even though more than 200 genes have been identified with mutations that cause retinal degeneration in humans, including forms of RP, it is estimated that the genes involved in approximately 35 % of AR retinal dystrophies remain unknown [28].

**Fig. 1:**
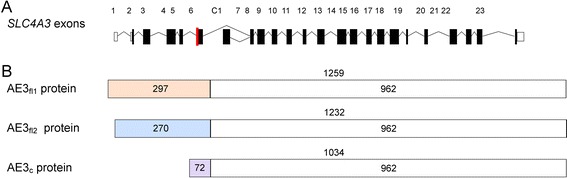
Genomic structure of the human *SLC4A3* gene and AE3 protein isoforms. **a** The three isoforms of *SLC4A3* are created by alternative splicing of exons 1–6 and C1, and by alternative splicing of exon 6 (*red*). **b** AE3_fl1_ and AE3_fl2_ proteins are created from alternative splicing of exon 6 and differ only with regards to the presence and absence respectively of 27 amino acids. AE3_c_ is created by alternative splicing of exon C1 and the first 72 amino acids are completely different than the full-length isoforms (*grey*). All three isoforms are identical over the 962 amino acids at the C-terminal end

Two main isoforms of *SLC4A3* have been described in humans: a full-length (*SLC4A3*_fl1_) isoform comprised of one non-coding (5’UTR) and 22 coding exons and a cardiac (*SLC4A3*_c_) isoform with 18 coding exons [4, 29]. These alternative transcripts differ at the 5’ end (Fig. 1). Alternative splicing of exon six of *SLC4A3*_fl1_ results in a further isoform (SLC4A3_fl2_) that differs by 81 bp. *SLC4A3*_fl1_ (Genbank: NM_201574.2) encodes a 1259 amino acid protein (Fig. 1), and this is the isoform referred to throughout the remainder of this manuscript. *SLC4A3*_fl2_ (GenBank: NM_005070.3) is the shorter version and encodes a protein of 1232 amino acids. In *SLC4A3*_c_ exon C1 replaces exons one to six of the full-length transcripts, and encodes a smaller protein product of 1034 amino acids [29]. A rare variant in the *SLC4A3* gene, Ala867Asp, has been associated with idiopathic generalized epilepsy (IGE) in humans, with carriers exhibiting an increased risk of developing IGE [30], but *SLC4A3* has not been implicated in human retinal disease. Evidence from the mouse and canine disease models suggest the *SLC4A3* gene is an excellent candidate for human retinal degeneration. We therefore screened *SLC4A3* in a cohort of human patients with predominantly recessive retinal degeneration currently lacking a molecular diagnosis in order to determine whether mutations in this gene cause a significant proportion of retinal degeneration in humans.

## Materials and methods

### Study subjects

The recruitment of all patients was part of a study protocol that adhered to the tenets of the Declaration of Helsinki and had received approval from the Moorfields Eye Hospital Research Ethics Committee. Written, informed consent was obtained from all participants prior to their inclusion in this study with parental written consent provided on behalf of any minors involved.

Two hundred affected unrelated individuals ascertained from the clinics of Moorfields Eye Hospital were evaluated for *SLC4A3* variants. Of these, 192 probands were affected with progressive retinal degeneration, consistent with a diagnosis of either retinitis pigmentosa or cone-rod dystrophy with presentation in adulthood (during or after the second decade). Eight additional patients with various forms of retinal degeneration were also selected for screening. In each of these 8 cases, autozygosity mapping previously conducted at UCL Institute of Ophthalmology (UCL, London) had identified large regions of homozygosity that included the genomic region containing *SLC4A3*, amongst other genes (Additional file 1). All 200 probands were assessed by AW, ATM or MM as part of their management in specialist inherited retinal clinics. Diagnosis was determined through a patient’s presenting history, clinical examination, retinal imaging including autofluorescence imaging and optical coherence tomography. Electroretinography was performed in those patients with milder disease (it is not informative in severe cases). Probands with a clinical history or signs suggestive of syndromic retinal dystrophy such as Usher (I, II or III), Bardet Biedl, Refsum disease, Joubert syndrome or Senior Loken syndrome for example were not included in this cohort. The family history was compatible in all cases with autosomal recessive inheritance. Simplex or male siblings in which X-linked retinal dystrophy was a possibility were screened for mutations in RP2 and RPGR (including ORF15) and were negative. Other candidate gene screening had been performed on a subset of probands as part of other similar projects, but had not had screening for all known retinal degeneration genes. Many probands, for instance, had been sequenced as part of a study of USH2A-related retinal dystrophy and those that were positive for *USH2A* mutations were excluded from this cohort. To our knowledge none of the patients had any other complications or symptoms, including epilepsy, however the possibility that such symptoms were undiagnosed or verbally unreported by the patient cannot be excluded. Normal healthy control samples were not collected as part of this study. Instead, exome variant data available for >46,000 individuals through the Exome Aggregation Consortium (ExAC) [31] was used for control data.

### Sequencing

Primers for amplification and sequencing of *SLC4A3* exons (Additional file 2), based on the known transcripts *SLC4A3*_fl1_ (GenBank: NM_201574.2), *SLC4A3*_fl2_(GenBank: NM_005070.3) and *SLC4A3*_c_ [29] were designed with Primer3 [32]. *SLC4A3* exons were amplified by PCR using HotStarTaq Plus DNA Polymerase (Qiagen) in genomic DNA. PCR products were purified using PCRμ96 filter plates (Millipore). Amplification products were sequenced using BigDye Terminator v3.1 (Applied Biosystems), and sequence product was purified using the Montage SEQ96 Cleanup Kit (Millipore), then run on an ABI 3730 Genetic Analyzer. Sequence traces were assembled, analysed and compared with the human reference sequence (GRCh37) using the Staden Package [33].

### Variant pathogenicity analysis

The potential pathogenicity of variants identified was assessed with various bioinformatics tools (Additional file 3). The ExAC Browser was used to determine whether any of the human variants discovered were novel and to determine allele frequencies of those variants previously identified. SIFT [34], PolyPhen2 [35] and PMut [36] were used to assess potentially pathogenic variants. A splice site prediction tool, NNSPLICE0.9 [37, 38], was used to identify any variants that may affect splicing.

## Results

Sequencing of all known exons and intron-exon boundaries of *SLC4A3* in 200 patients revealed 50 SNP variants (Additional file 3) carried by at least one individual, but no small insertions or deletions within exons, when compared with the human reference sequence (GRCh37). Of these, 21 were located in exons, 23 in introns, four in the 5’-UTR and two in the 3’-UTR. The majority (*n* = 42) have previously been identified and have entries in the dbSNP database, and 31 are present in the Exome Aggregation Consortium (ExAC) database. Copy number variants, such as large deletions and insertions within introns, or affecting upstream promoter sequences, were not assessed in this study.

**Fig. 2:**
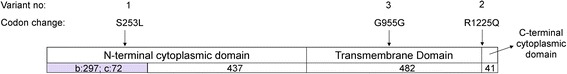
Location of potentially pathogenic variants identified in RP patients on the AE3 protein. The number of amino acids that constitute each protein domain are indicated. SNPs_2 and _3 are located in the domains shared by the full-length (b) and cardiac (c) isoforms. SNP_1 is located in the N-terminal cytoplasmic domain unique to the full-length isoform (*grey*). SNP = single nucleotide polymorphism

After elimination of variants that were unlikely to be pathogenic due to allele frequency in the population (>0.03) and/or prediction of pathogenicity, three rare variants remained that were predicted to affect exon splicing and/or change the amino acid sequence of the protein (Table 1). SNP_1 affects only the full-length *SLC4A3* isoforms, while SNPs_2 and _3 affect the full-length and cardiac isoforms (Fig. 2). Three patients from the panel of 192 AR RP cases carried one of these three variants in the heterozygous state.

**Table 1:** Rare variants predicted to be potentially deleterious

SNP^a^ ID	Genomic Location	A1^b^	A2^c^	Possible effect on splice sites	Nucleotide change	Codon change	Reference SNP (rs) ID	Predicted to be Pathogenic by^d^
1	2:220494940	C	T	None	c.758C>T	S253L	rs36068948	PP
2	2:220505656	G	A	Introduction of acceptor site (0.00-0.81)	c.3674G>A	R1225Q	rs150952379	PP, S, PM, N
3	2:220502903	G	A	Loss of acceptor site (0.45-0.00)	c.2865G>A	G995G	rs387907534	N

^a^SNP = single nucleotide polymorphism

^b^Allele 1 = Reference Allele

^c^Allele 2 = Minor/Variant Allele

^d^PP = PolyPhen; S = SIFT; PM = PMut; N = NNSPLICE

**Fig. 3:**
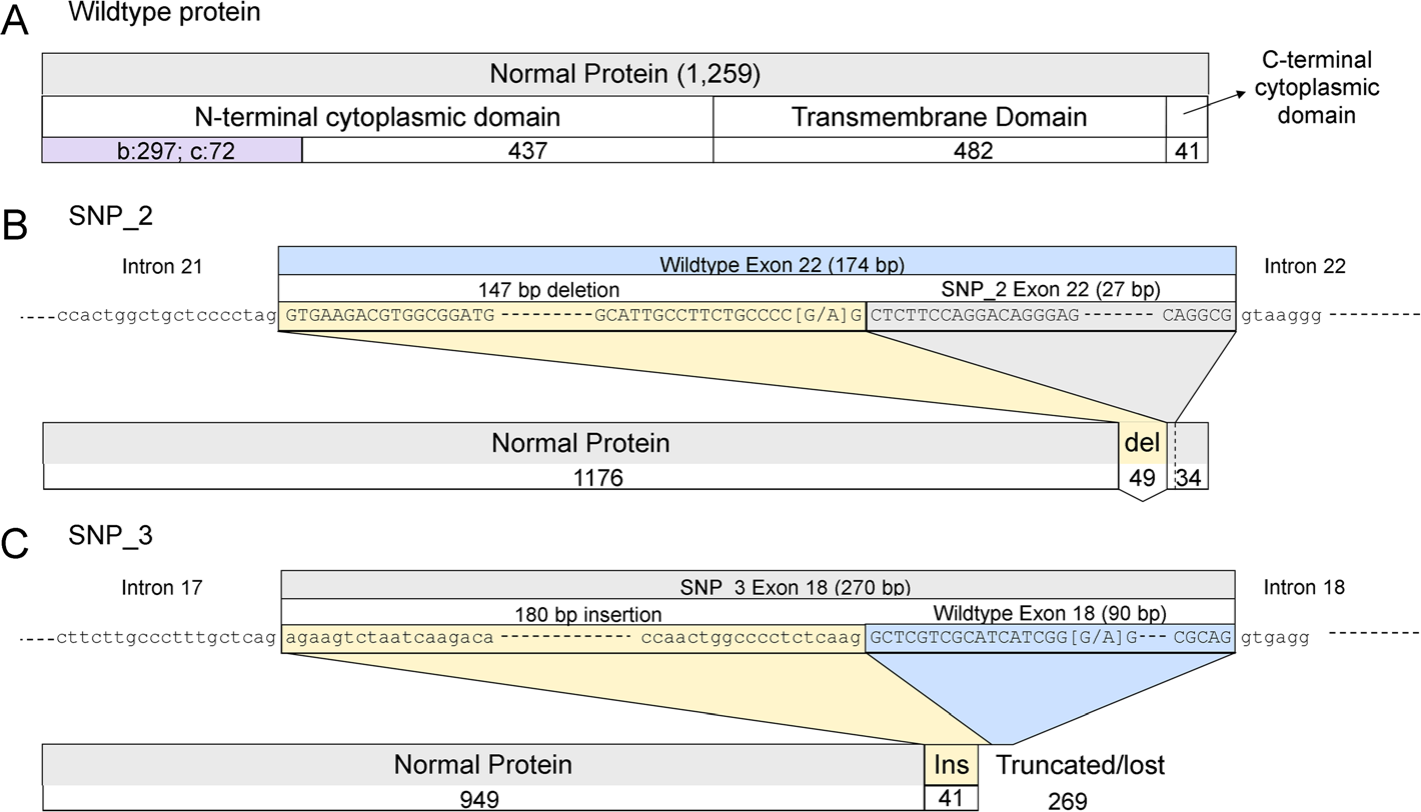
Predicted splicing effects of two exonic variants on the AE3 protein. Graphical representation of the wildtype human AE3 protein and the predicted splicing effects of exonic variants on the protein. The numbers of amino acids are indicated, and the nucleotides affected by the variants are flanked by square brackets. **a** Normal full-length (AE3fl1, 1259 amino acids) protein. **b** SNP_2 may introduce an acceptor site resulting in the reduction in the size of exon 22 from 174 to 27 bp. This results would be an in-frame deletion of 49 amino acids near the C-terminus of the protein, and a final protein 1210 amino acids in size. **c** SNP_3 may remove an acceptor site resulting in an increase in the size of exon 18 from 90 to 270 bp, which would result in an insertion of 41 amino acids (Ins), a premature termination codon, and the loss of 269 amino acids at the C-terminus. SNP = single nucleotide polymorphism

SNPs_1 (c.758T>C, p.S253L) and _2 (c.G3674A, p.R1225Q) are non-synonymous SNPs that result in amino acid changes that are predicted to be pathogenic by at least one of the in silico prediction tools (PolyPhen, SIFT and PMut; Table 1). SNP_1 is predicted to affect the full-length isoforms only and SNP_2 the full-length and cardiac isoforms. SNP_2 is also predicted to affect splicing of all three isoforms of the protein, with the potential to introduce a new acceptor site (Fig. 3b). This would result in the deletion of 49 amino acids near the C-terminus of the protein. SNP_3 (c.G2865A, p.G955G) is predicted to affect exon splicing, presumably, as it is located 18 bp 3’ of the acceptor spice site, by affecting an exon splicing enhancer. Due to its location in exon 18, SNP_3 would affect all three isoforms of the protein. It is predicted to remove an existing acceptor site (Fig. 3c), resulting in a reading frame shift, early termination codon, and loss of 269 amino acids.

These three rare variants all have potentially pathogenic, loss-of-function effects on the AE3 protein. Each variant was only detected in a single patient in the heterozygous state (Table 2). All three variants were also heterozygous in a number of controls: SNP_1 in 146/56,676 controls, SNP_2 in 93/60,187 controls and SNP_3 in 8/46,201 controls. SNP_1 and SNP_2 were each homozygous in one control. In addition no patients carried more than one *SLC4A3* variant.

**Table 2:** Variant frequency in patient and control cohorts

Variants^a^	200 Patient Panel				ExAC			
SNP_ID^b^	A1/A1	A2/A1	A2/A2	A2 Freq	A1/A1	A2/A1	A2/A2	A2 Freq
1	199	**1**	**0**	0.003	56,529	**146**	**1**	0.001
2	199	**1**	**0**	0.003	60,093	**93**	**1**	7.89x10^−4^
3	199	**1**	**0**	0.003	46,193	**8**	**0**	8.66x10^−5^

^a^A1 = Reference Allele, A2 = Minor/Variant Allele, depicted in Bold

^b^SNP = single nucleotide polymorphism

## Discussion

Despite the association of aberrant *SLC4A3* with retinal degeneration in two model species, mouse and dog, similar studies investigating possible involvement of the gene in human retinal degeneration have not been reported to date. Of the 14 genes reported to be involved in canine retinal degeneration [39], 11 have also been implicated in human retinal disease [8]. It is therefore possible, if not probable, that mutations in *SLC4A3* could cause retinal disease in humans. We report here the first such study to screen human patients with retinal disease for potentially pathogenic mutations in the *SLC4A3* gene.

The full-length isoforms of AE3 are expressed predominantly in the brain, but it is also found in the gut, kidney, heart and Müller cells of the retina [1, 2, 29, 40, 41]. The cardiac isoform is expressed predominantly in the heart and also in the horizontal neurons of the retina [1, 2, 41]. The structure of the protein is thought to be similar to another family member, SLC4A1 (AE1). SLC4 proteins are made up of three structural domains. At the N-terminus there is a hydrophilic, cytoplasmic domain of between 400 and 700 amino acids, followed by a hydrophobic, polytopic transmembrane domain of approximately 500 amino acids, comprising up to 14 transmembrane spans, and lastly a cytoplasmic domain of between 30 and 100 amino acids at the C-terminal end [1, 40].

While *SLC4A3* has not been directly linked with disease in humans, there is evidence that indicates the protein is vital to normal brain and cardiac function. The p.Ala867Asp variant confers susceptibility to idiopathic generalised epilepsy [30]. This variant results in decreased AE3 transport activity that could cause abnormal intracellular pH and changes in cell volume, which in turn may promote neuron hyperexcitability and the generation of seizures [42]. The p.Ala867Asp variant was not identified in any of the cases screened, nor has epilepsy been reported in the current patient cohort. Nevertheless, we cannot exclude the possibility that epilepsy is present, but has simply not been diagnosed, or reported to us, in our patient cohort. Hentschke et al. described a *SLC4A3* knockout mouse that appeared healthy, but had a reduced seizure threshold when exposed to bicuculline, pentylenetetrazole or pilocarpine, and increased seizure-induced mortality [43]. In addition, AE3c has a key role in myocardial intracellular pH recovery from alkaline loads [44]. While the loss of AE3 alone has no known adverse effects on the heart, the combined loss of AE3 and sodium/potassium/chloride transporter 1 (NKCC1, aka SLC12A2) impairs cardiac function [45]. Similarly, loss of AE3 in the TM180 transgenic mouse (with a Glu180Gly substitution in the α-tropomyosin gene) led to more rapid decompensation and heart failure than the TM180 mouse alone [46].

Screening of the exons of *SLC4A3* in DNA samples from 200 patients with retinal degeneration resulted in the identification of three rare variants that are predicted (by *in silico* methods) to be potentially deleterious. Three individuals with AR RP carry one copy of the minor allele at one of these loci (Table 2), i.e. are heterozygous. The variants are therefore insufficient to cause AR RP in isolation. None of the heterozygous individuals were found to carry more than one rare *SLC4A3* variant and do not, therefore, appear to be compound heterozygotes. However, in this study we did not evaluate non-coding regions of the gene or exclude the possibility of partial gene deletions of the other allele. In the same way that the p.Ala867Asp variant predisposes patients to epilepsy it is possible that one of the variants identified in the present study predisposes patients to retinal degeneration, perhaps only in conjunction with a mutation at another location. Alternatively, a heterozygous mutation could modify the penetrance or age of onset of retinal degeneration caused by a mutation at another location. Whole genome or exome sequencing could identify a second hit at another locus, but is outside the scope of the present study.

All three rare variants were also seen in the control datasets. SNP_1 (c.758T>C, p.S253L) and SNP_2 (c.3674G>A, p.R1225Q) were found in the homozygous state in a single control exome each, as well as in the heterozygous state in multiple exomes, at a frequency of 7.89x10^−4^ and 8.66x10^−5^ respectively. SNP_3 (c.2865G>A) was found in the heterozygous state only at a frequency of 8.66x10^−5^. The relatively high frequency of SNP_1 in the control exomes and the observation that it was predicted to be potentially pathogenic by only one of the *in silico* prediction tools suggests the variant is unlikely to play a role in retinal disease. SNP_2 has a relatively high allele frequency and occurs in the homozygous state in the control cohort. This suggests that this rare variant is also unlikely to cause disease in isolation, even though this variant was predicted to be potentially pathogenic by all four *in silico* prediction tools. Finally, SNP_3 has a very low allele frequency in the control exome data (8.66x10^−5^), but is predicted to affect exon splicing only. The SNPS predicted to affect exon splicing (SNPs_2 and _3; Fig. 3) could be further investigated through analysis of mRNA transcripts, if the transcripts are detected in easily accessible tissue such as blood or buccal cells. However, as we have previously found that canine *SLC4A3* mRNA is undetectable in blood or buccal cells (unpublished data), human *SLC4A3* mRNA is also likely to be undetectable and this was not pursued. In vitro assays could be useful to assess pathogenicity of SNP variants. However, it is difficult to select which variants to test, and the assay to use until more convincing evidence is presented that *SLC4A3* is involved in human retinal disease. Such a study would therefore be premature at this stage.

While it appears that the variants in *SLC4A3* identified in this study are unlikely to cause AR RP in the cohort screened in isolation, we cannot fully exclude these variants or the gene as a candidate for retinal degeneration. Potentially pathogenic rare variants in intronic regions or upstream elements that were not screened for mutations by sequencing could affect exon splicing or regulation of gene expression. In addition, it is possible that disease-causing mutations in this gene are extremely rare and essentially private mutations affecting only one or two affected individuals, as is increasingly the case in consanguineous families with a recessive condition, and that these individuals have yet to be screened. For example, a *PRCD* gene mutation (p.C2Y) was reported to cause PRA in dogs and RP in one person. However, no other disease-causing mutations were found in *PRCD* in a further 1240 RP patients screened [47]. Disease-causing mutations in this gene are therefore exceedingly rare in the general population. However, a second pathogenic mutation in *PRCD* has since been identified in an isolated Muslim Arab village in Northern Israel; This founder mutation was homozygous in all 18 RP-affected individuals, but not in any of the 28 unaffected family members [48]. While the identification of cases caused by mutations in other RP genes has been accelerated by the use of exome sequencing, this has not been the case for *PRCD* [49, 50]. Finally, it is also possible that mutant *SLC4A3* actually causes a syndromic or non-syndromic form of retinal degeneration in humans that would not be clinically classified as RP. Little is known about the phenotype associated with GR_PRA1 in dogs beyond ophthalmoscopic observations. Clinically the fundus appears identical to other forms of PRA and histological and detailed ERG analysis has not been reported. In addition, the age of onset is difficult to define, although the age at which dogs with GR_PRA1 are diagnosed is typically 6–7 years [4]. This is suggestive of a relatively late-onset condition, a hypothesis consistent with the findings in the *SLC4A3* knockout mouse in which a selective inner retina defect is followed by photoreceptor degeneration at eight months [3]. Given the late onset of clinical signs in the dog and mouse models, early onset degenerations such as LCA are unlikely to be caused by variants in *SLC4A3*. Alvarez and colleagues concluded that their results in the knockout mouse linked aberrant *SLC4A3* to vitreoretinal degeneration [3]. While there are similarities to vitreoretinal disorders, the data presented for the mouse phenotype is not pathognomonic of a particular human phenotype. In addition, vitreoretinal disorders are rare and DNA samples may be difficult to obtain. The other abnormalities reported by Alvarez and colleagues were altered ERG and abnormal retinal vessels, and these clinical features are shared by many vitreoretinal and retinal degenerations. Currently there is no evidence to suggest a human phenotype that would be more likely to be caused by variants in *SLC4A3* than AR RP, but other phenotypes should not be excluded from future studies.

## Conclusions

Aberrant *SLC4A3* has been shown to cause retinal disease in the mouse and dog, making the gene a strong candidate for human retinal disease. Three rare variants predicted to be potentially pathogenic were identified in the *SLC4A3* gene in a AR RP cohort, however all three variants were present in the heterozygous state, and therefore not disease-causing in isolation. Nevertheless, we could not discount the possibility that these variants have some role in the disease that we have yet to decipher. The *SLC4A3* gene remains an excellent candidate gene for human retinal degeneration and the variants identified will help to build a picture of its potential contribution.

### Ethics approval and consent to participate

The recruitment of all patients was part of a study protocol that adhered to the tenets of the Declaration of Helsinki and had received approval from the Moorfields Eye Hospital Research Ethics Committee. Written, informed consent was obtained from all participants prior to their inclusion in this study with parental written consent provided on behalf of any minors involved.

### Consent for publication

Not applicable.

### Availability of data

The datasets supporting the conclusions of this article are included within the article and its Additional files 1, 2 and 3.

## Additional files

Additional file 1: Molecularly unsolved patients with autozygosity data indicating a homozygous region containing SLC4A3. In eight of the individuals studied, previous autozygosity mapping had identified loci associated with retinal disease. Here we list the sizes of the loci and the number of genes in each. (PDF 16 kb) Additional file 2: PCR and Sequencing primers and experimental conditions. Description of the thermal cycling parameters for PCR amplification and Sanger sequencing, and primer sequences (including PCR product size and genomic location of exons). (PDF 210 kb) Additional file 3: All sequence variants identified. Description of all sequence variants identified, including genomic location, nucleotide and amino acid change, predicted effect of splice sites, and SNP identifier. (XLSX 20 kb)

## Abbreviations

AE3: anion exchanger 3
AR: autosomal recessive
IGE: idiopathic generalised epilepsy
LCA: leber congenital amaurosis
PRA: progressive retinal atrophy
RP: retinitis pigmentosa
SLC4A3: solute carrier family 4, member 3
SNP: single nucleotide polymorphism
UTR: untranslated region
XL: X-linked
