# Supplementary material for: Investigation of SLA4A3 as a candidate gene for human retinal disease
Source: J Negat Results Biomed. 2016 May 23;15:11. doi: 10.1186/s12952-016-0054-z (PMC4876561; doi:10.1186/s12952-016-0054-z)
Supplement: Additional file 1: — Molecularly unsolved patients with autozygosity data indicating a homozygous region containing SLC4A3. In eight of the individuals studied, previous autozygosity mapping had identified loci associated with retinal disease. Here we list the sizes of the loci and the number of genes in each. (PDF 16 kb) [file 12952_2016_54_MOESM1_ESM.pdf]

Additional File 1: Molecularly unsolved patients with autozygosity data indicating a homozygous region containing *SLC4A3*

Patient ID	Diagnosis	Homozygous region containing *SLC4A3*			Overview of other genomic regions identified by autozygosity mapping		
		Size (Mb)	No of genes	Retinal genes*	Size (Mb)	No of genes	Retinal genes*
1	AR RP	28.78	193	3	19.74	74	0
2	AR RP	19.24	158	4	88.69	503	5
3	Rod monochromacy	9.42	39	1	280.7	2218	21
4	Recessive retinal dystrophy	22.59	182	3	222.93	2010	22
5	AR RP	36.36	271	3	94.87	1271	20
6	AR RP	13.91	99	1	172.98	1334	17
7	AR RP (pseudo dominant)	14.16	93	1	369.7	1774	20
8	cone-rod dystrophy	16.07	102	3	34.38	281	1

* Genes previously associated with retinal disease in humans
