# Supplementary material for: Investigation of SLA4A3 as a candidate gene for human retinal disease
Source: J Negat Results Biomed. 2016 May 23;15:11. doi: 10.1186/s12952-016-0054-z (PMC4876561; doi:10.1186/s12952-016-0054-z)
Supplement: Additional file 2: — PCR and Sequencing primers and experimental conditions. Description of the thermal cycling parameters for PCR amplification and Sanger sequencing, and primer sequences (including PCR product size and genomic location of exons). (PDF 210 kb) [file 12952_2016_54_MOESM2_ESM.pdf]

Additional File 2: PCR and Sequencing primers and experimental conditions

Table 1 PCR Reaction Thermal Cycling

	Temperature	Duration	Cycles
Initial Denaturation	94°C	10 min	
Denaturation	94°C	1 min	X35
Annealing	58°C	1 min	
Extension	72°C	2 min	
Final Extension	72°C	10 min	

Table 2 Sequencing Reaction Thermal Cycling

	Temperature	Duration	Cycles
Initial Denaturation	96°C	2 min	
Denaturation	96°C	10 sec	X25
Annealing	50°C	5 sec	
Extension	60°C	4 min	

Table 3 Primers

Exon	Forward Primer Sequence (5'-3')	Reverse Primer Sequence (5'-3')	Product Size (nucleotides)	Additive, Alteration[1]	Exon Genomic Location[2]
1A	AGGCAAGGCTGTGGTAGAAAT	TCTCGCAGCGCAGAGC	444	Q, 98	220,491,773
1B	CCCGCTCCCATTGTGC	CCGAGACCCAGGTGAGAAG	443	Q, 98	220,491,773
2	GGGGATTGTGGGAATATCG	GGGCTACGGATCTGGAAAATA	849	Q, 98	220,482,089
3	CAGATCCGTAGCCCTCTCTCT	CACAGAGTCCAGCACAGTGTC	498		220,492,924
4	GACTAGGGTGCCCTTGTTTG	CAACACGCACTCCTCTCCTC	464		220,493,757
5	AGGGGTACAGAGAGGAGGAGA	GTCACTCTCTCCTGGGGAACT	484		220,493,187
6	GAGAGGGTGAGGAGAAAGGTG	AGAGCCTCTCCAAGGTCACTC	475		220,494,677
6b	ACCCTGAGAAAAGAGGAGCAG	AGAGGACTCAGCCCTTAGCAG	660		220,494,523
7	ATGGCCTGTTGGTGACTGTAG	GTTCAGCTCCACGAACACCT	430		220,496,572
8	ATCCTTCGCAGGAAGAAGAAG	GAGAGTGTTCCTGGGAACTGA	457		220,496,788
9	GTCAGTCAACAAATGGCTTCC	CTCTGTCACTTGTGGCATTCA	489		220,497,353
10	TGAATGCCACAAGTGACAGAG	CAACCCACTCAGTGAAGTCGT	447		220,497,821
11	TGCAGTACCTGCCTACTCTGG	CAGGGGACTACGAAAGAAAGG	402		220,498,859
12	TTCTTTGCGCAGTTTACAACC	ACTGTGGACAATGAGGGACAG	373		220,499,378
13	CCTGTAGCTCAGTGACCCAAC	GATCAGGGATGCAAAGGACTC	430		220,499,899
14	GAGAGCGTGAACAGACCAAAG	ATTCCCTGATCCAGCATCTCT	620		220,500,755
15	GTCACTCTAAGGGGCTGCTCT	CAGAACTAGGTGGGGGTAGGA	499		220,500,908
16	ATTCTCCCTCTTCCTCGGAGT	CTAGAACCCCTCACTCCCAAC	430		220,501,301
17	CACAGGTGTACTGATGACGATG	AAGAAGACACCCAGGGAAGAG	466		220,502,227
18	CTCTTCCCTGGGTGTCTTCTT	TCCACTGGGAAAGTTGAGTTC	423		220,502,672
19	TTCATTTCCCTGCCCTCTATT	CCCAGCACCCTGATACTGATA	470		220,503,232
20	TGAAAGAAGAAGCTGGCAGTC	ACACAGCACATTGGGAGATTC	496		220,504,538
21	TCCTTCTCTTGTTCAGGGTGA	CCTTCATATTCATCCCATCCA	452		220,504,964
22	TGAAGGAGAAGCTGTGACCTG	GGCCTTTACCTGGACTAGCAC	499		220,505,410
23	AGGAGGAGCTGGAGAATGG	CTCAAACCTCTCCCTGGTTTT	500		220,506,302
C1	GGGAGGGCGTGTTTACAGA	TTCGCTCCCTTCCTCCTGT	489		220,495,736
Internal primers for sequencing only					
2int	CTGTCCCGGGCGTGGCTGGG				

[1] Q = Q Solution, 98 = Initial denaturation and denaturation steps at 98°C.

[2] Build GRCh37
